# Muscle Adaptation to Varying Oxygen Concentrations During Weaning in Rats

**DOI:** 10.7759/cureus.92807

**Published:** 2025-09-20

**Authors:** Takumi Saito, Yukino Mori, Issei Sugimoto, Yugo Kimura, Ikue Kondo, Atsunori Itagaki, Sangun Lee

**Affiliations:** 1 Graduate School of Health Sciences, Aomori University of Health and Welfare, Aomori, JPN; 2 Department of Rehabilitation, Matsuda Hospital, Sendai, JPN; 3 Department of Physical Therapy, Aomori University of Health and Welfare, Aomori, JPN; 4 Department of Rehabilitation, Aomori Prefectural Central Hospital, Aomori, JPN; 5 Department of Rehabilitation, National Hospital Organization (NHO) Kamaishi National Hospital, Kamaishi, JPN; 6 Department of Rehabilitation, Edogawa Hospital, Edogawa, JPN; 7 Department of Physical Therapy, Tokyo Metropolitan University, Arakawa, JPN

**Keywords:** muscle development, muscle fiber, oxidative stress, oxygen concentration, weaning phase

## Abstract

Introduction: This study examined the effects of different oxygen concentrations on muscle tissue and oxidative stress dynamics in rats during the weaning period and aimed to reveal the specificity of development during the growth stage.

Methods: Thirty three-week-old male Wistar rats were randomly divided into five groups: control (group III), 10% oxygen (group I), 15% oxygen (group II), 40% oxygen (group IV), and 80% oxygen (group V). After the experiment, the soleus and plantaris muscles were stained with an adenosine triphosphatase stain. The percentage of muscle fiber types and cross-sectional area (CSA) was calculated, and oxidative stress was measured using blood serum. Scheffé's test was used for analysis. Statistical significance was set at p<0.05.

Results: The percentage of type Ⅰ fibers in the soleus muscle was significantly higher in groups I, II, and IV compared to group III. CSA in the soleus muscle were significantly smaller in groups I, II, IV, and V than in group III. Conversely, CSA in the plantaris muscle were significantly lower in groups I and II compared to group III, whereas groups IV and V exhibited significantly greater CSA than group III. No significant differences in oxidative stress levels were observed among the groups.

Conclusion: Exposure to different oxygen concentrations induced muscle fiber type-specific adaptive responses during development, without promoting excessive reactive oxygen species production. Therefore, our results suggest that varying oxygen concentrations require consideration for applications in developmental physiology, pediatric rehabilitation, and preventive medicine.

## Introduction

The muscle fiber is classified into type I and II fibers based on contraction speed and energy metabolism characteristics [1]. Type Ⅰ fibers, which contain abundant mitochondria and myoglobin, primarily generate adenosine triphosphate (ATP) through oxidative phosphorylation [1]. Additionally, the capillary density in type I fibers exceeds that of type II fibers [1], which allows type Ⅰ fibers to adapt effectively for oxygen (O₂) uptake. Therefore, changes in O₂ concentration influence muscle fibers differently depending on their metabolic characteristics.

Under hypoxic conditions, muscle tissues enhance O₂ diffusion to muscle cells by increasing the percentage of type Ⅰ fibers and decreasing the cross-sectional area (CSA) [2-4]. Conversely, exposure to high O₂ concentrations also increases the percentage of type Ⅰ fibers [5] and contributes to the recovery from unloading-induced muscle atrophy [6] and muscle injuries [7]. Thus, both hypoxia and hyperoxia increase the proportion of type Ⅰ fibers, although their impacts on CSA and energy metabolism differ. However, the effects of varying O₂ concentration characteristics on muscle morphology during the weaning phase have not been clarified.

Reactive oxygen species (ROS) play an essential role in immune function within the body [8]. Approximately 2-5% of O₂ uptake converts into ROS, and antioxidant systems regulate oxidative stress to maintain homeostasis [9]. O₂ toxicity and ischemia enhance electron leakage within cells [10,11]. Excessive ROS production impairs mitochondrial function, increases glycolytic metabolism, and reduces the percentage of type Ⅰ fibers [12,13]. Particularly, oxidative stress dynamics exhibit heightened sensitivity to O₂ concentration changes during the weaning phase, when the immune system and associated organs remain immature [14].

This study examined the effects of hypoxia and hyperoxia on muscle tissue and oxidative stress dynamics in weaning rats, aiming to gain insights into muscle fiber development in response to varying O₂ concentrations.

## Materials and methods

Experimental animals

Thirty three-week-old male Wistar rats (CLEA Japan, Inc., Japan) were used in this study and randomly divided into five groups: control (group III; n=6), 10% O₂ (group I; n=6), 15% O₂ (group II; n=6), 40% O₂ (group IV; n=6), and 80% O₂ (group V; n=6). Each pair of rats was housed in one cage under controlled conditions: room temperature of 23±1℃, humidity of 55±5%, and a 12-hour light-dark cycle (7:00 PM to 7:00 AM). All animals had free access to food CE-2 (CLEA Japan, Inc., Japan). This study was approved by the Animal Care and Use Committee of Aomori University of Health and Welfare (approval number: 22003) and was conducted in accordance with the guidelines for animal experiments at Aomori University of Health and Welfare.

Experimental protocol

The experimental setup included a monitor sensor (OM-25MP11, Taiei Engineering Co., Ltd., Japan), medical O₂ and nitrogen gas tubes, and a sealed box containing the animals. O₂ concentration levels were controlled using the SSJ-OX-E device (Yamato Sangyo Co., Japan). A previous study reported that exposure to 10% O₂ increased the percentage of type Ⅰ muscle fibers [2], whereas 15% O₂ showed no significant effect [15]. Conversely, exposure to 36% O₂ under altered atmospheric pressure conditions increased the type Ⅰ fiber proportion [5], whereas 80% O₂ reduced it [13]. Hypobaric environments have been linked to dysfunctions in cardiovascular, respiratory, and reproductive systems [16,17], whereas hyperbaric environments may cause O₂ toxicity and central nervous system disorders [18,19]. Based on this background, this study used normobaric conditions with 10%, 15%, 40%, and 80% O₂ concentrations. Each group was exposed to the assigned O₂ level for one hour daily (9:00 AM to 10:00 AM) over a four-week period.

Muscle evisceration

The body weight of each animal was measured with a digital scale before and after the experiment. An intraperitoneal injection of a mixed anesthetic solution (0.5 mL per 100 g body weight) was administered. The anesthetic mixture included medetomidine hydrochloride (1.88 mL), midazolam (2 mL), butorphanol tartrate (2.5 mL), and normal saline (18.63 mL), totaling 25 mL. Under deep anesthesia, the soleus muscle (Sol) and plantaris muscle (PL) were carefully excised. These muscle tissues were embedded in Tissue-Tek® O.C.T. Compound (Sakura Finetek USA Inc., Torrance, CA, USA), rapidly frozen in isopentane, and stored at -80℃ for further analysis.

Oxidative stress and antioxidant capacity

Oxidative stress and antioxidant capacity in the plasma were evaluated by measuring reactive oxygen metabolites (d-ROMs) and biological antioxidant potential (BAP) following the experimental protocol [20,21]. Both d-ROM and BAP values were measured using the REDOXLIBRA analyzer (Wismerll Co., Ltd., Japan). Blood samples were collected from the tail vein after the final exposure session and centrifuged. The resulting serum was transferred into microtubes and stored at -80℃ until analysis.

Oxidative Stress (d-ROMs)

The d-ROMs test measures the colorimetric reaction of serum hydroperoxides. A 20 μL aliquot of serum was mixed with an acid buffer (pH 4.8) and subsequently transferred to a cuvette preloaded with dry chromogen for analysis. The sample was centrifuged, and the intensity of the color reaction was measured at a wavelength of 505 nm for five minutes. 

Antioxidant Capacity (BAP)

The BAP test measures the reduction of Fe3+ to Fe2+ by antioxidants present in the serum. A 50 μL aliquot of the iron-based chromogen solution (R2 reagent) was transferred into a thiocyanate solution and mixed. The color reaction was monitored using a photometer for three seconds. Subsequently, a 10 μL serum sample was added to the mixture and combined. The antioxidant capacity was determined by measuring the color change at a wavelength of 505 nm over three seconds.

Histochemical analysis

ATPase staining was performed using the method described by Padykula and Herman [22] to distinguish type I and II muscle fibers in the Sol and PL muscles. Samples were rapidly frozen in isopentane cooled with liquid nitrogen and sectioned at 10 µm thickness using a cryostat maintained below -20℃. Three serial sections were prepared per muscle, and ATPase staining at pH 10.4 was applied. The procedure included preincubation in a solution of 0.1 M sodium barbital and 0.18 M CaCl₂ (adjusted with HCl and NaOH) for 15 minutes, followed by a 45-minute incubation in the same buffer containing 50 mg of adenosine 5′-triphosphate (ATP; Oriental Yeast Co., Ltd., Japan). Sections were then washed three times in 1% CaCl₂, incubated for three minutes in 2% CoCl₂, washed eight times in 0.01 M sodium barbital, and incubated for one minute in 1% ammonia. Dehydration and mounting were performed according to standard histological methods. After staining, representative areas of each section were imaged with a digital microscope camera, and fiber type proportions were calculated. A total of 100 muscle fibers from three sections per sample were analyzed using the WinROOF 2021 software (Mitani Co., Japan).

Statistical analysis

Statistical analysis was performed using R Commander. Comparisons between two groups were made using paired t-tests, and differences among multiple groups were assessed using one-way analysis of variance followed by Scheffé's post hoc test. Statistical significance was set at p<0.05.

## Results

Body weight

**Figure 1:**
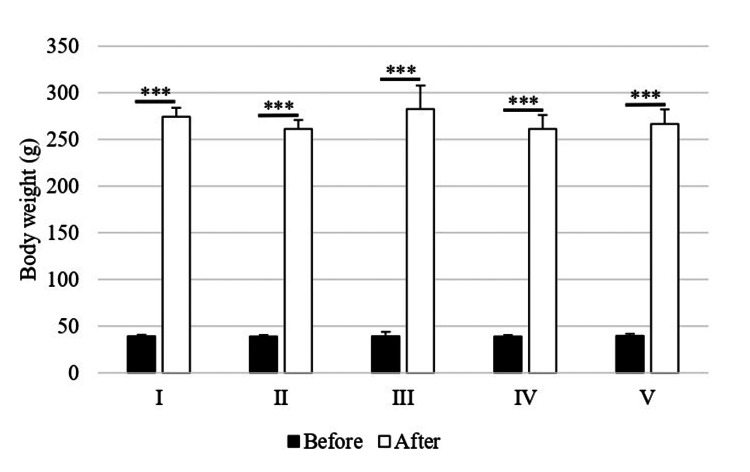
Body weight Comparisons between the two groups were made using paired t-tests, and differences between the groups either before or after the experiment were assessed using one-way analysis of variance followed by Scheffé's test. ***p<0.001 I: 10% O₂; II: 15% O₂; III: control; IV: 40% O₂; V: 80% O₂

No significant differences in body weight were observed between the groups either before or after the experiment. However, post-experiment body weights were significantly increased compared to pre-experiment values within each group: 703.1% for group I, 673.2% for group II, 724.4% for group III, 673.2% for group IV, and 674.7% for group V (all p<0.001) (Figure 1).

Muscle fiber type composition

**Figure 2:**
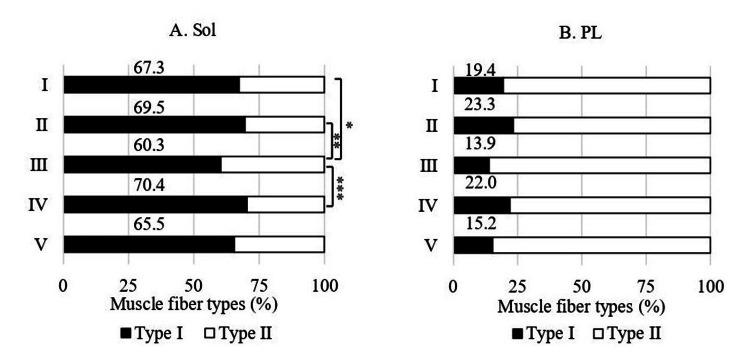
Muscle fiber type composition of the Sol and PL Statistical analysis were performed using one-way analysis of variance followed by Scheffé's test. *p<0.05; **p<0.01; ***p<0.001 Sol: soleus muscle; PL: plantaris muscle; I: 10% O₂; II: 15% O₂; III: control; IV: 40% O₂; V: 80% O₂

In the Sol, the proportion of type Ⅰ fibers followed the order: group III (60.3%)<group V (65.5%)<group I (67.3%)<group II (69.5%)<group IV (70.4%). The percentages in groups I, II, and IV were significantly higher than those in group III by 11.6% (p<0.05), 15.3% (p<0.01), and 16.7% (p<0.001), respectively (Figure 2A). In the PL, the type I fiber proportions followed the order: group III (13.9%)<group V (15.2%)<group I (19.4%)<group IV (22%)<group II (23.3%) (Figure 2B).

Muscle CSA

Muscle Total CSA

**Figure 3:**
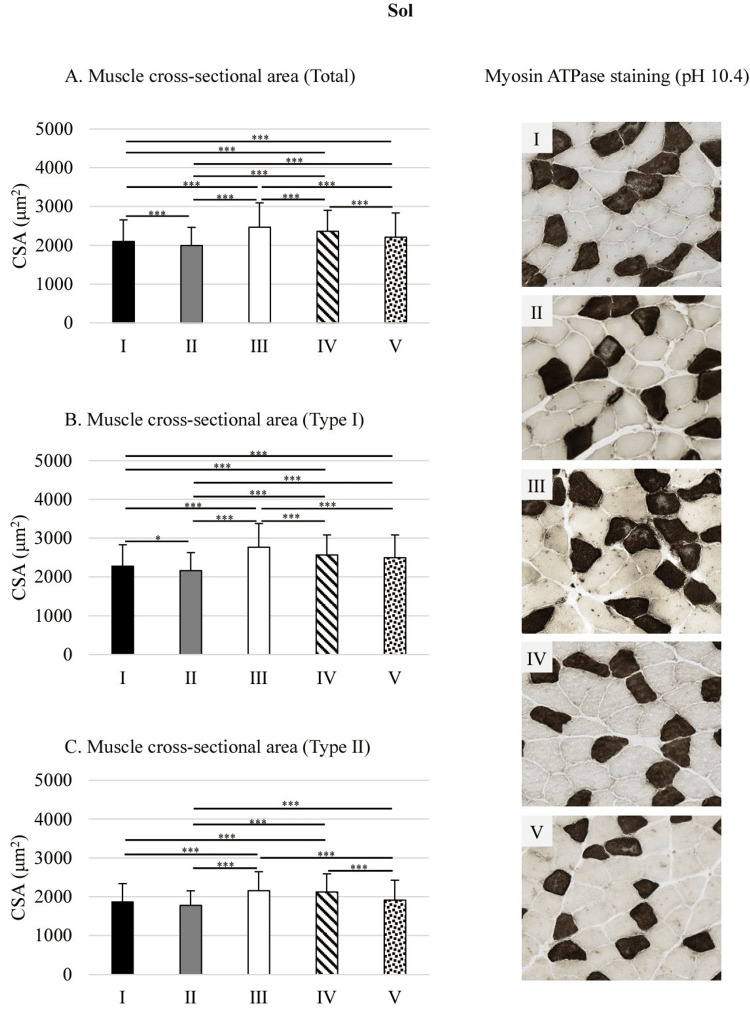
Muscle CSA in the Sol Statistical analysis were performed using one-way analysis of variance followed by Scheffé's test. *p<0.05; ***p<0.001 Sol: soleus muscle; CSA: cross-sectional area; I: 10% O₂; II: 15% O₂; III: control; IV: 40% O₂; V: 80% O₂

In the Sol, total CSA was significantly lower in groups I, II, IV, and V compared to group III by 14.7%, 19.1%, 4.4%, and 10.5%, respectively (all p<0.001). Under high O₂ concentration, group IV had a significantly greater CSA than group V by 6.8% (p<0.001). Under low O₂ concentration, group I showed significantly greater CSA than group II by 5.4% (p<0.001). When comparing high vs. low O₂ conditions, the CSA in groups I and II were significantly lower than in group IV by 10.8% and 15.4%, respectively (all p<0.001), and lower than in group V by 4.8% and 9.7%, respectively (all p<0.001) (Figure 3A).

**Figure 4:**
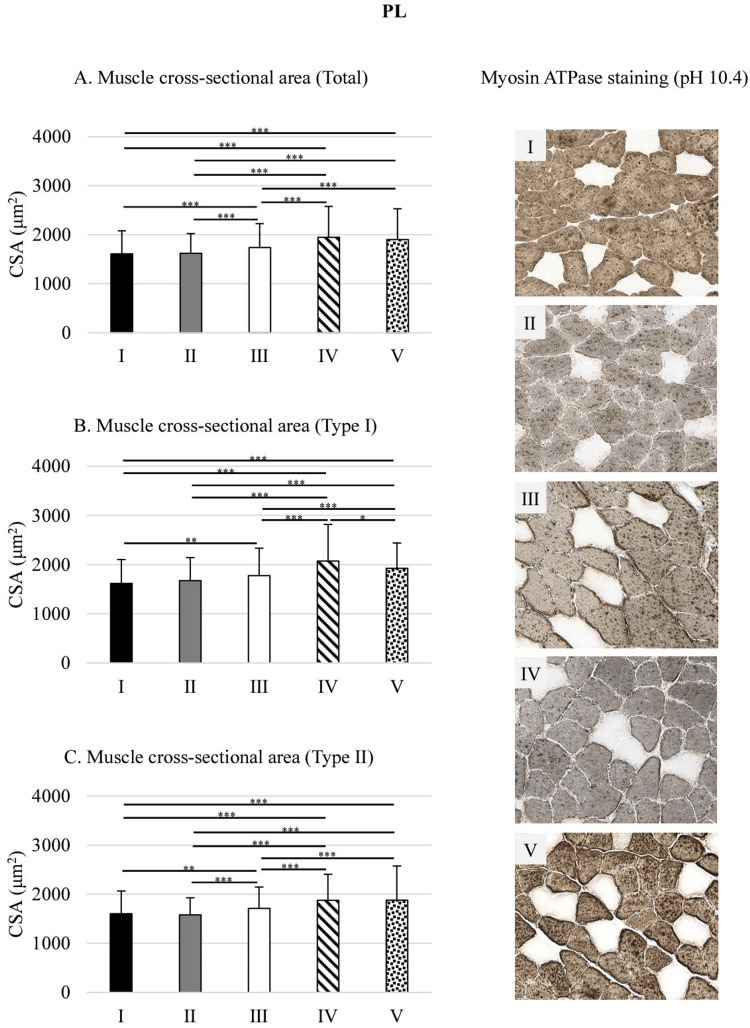
Muscle CSA in the PL Statistical analysis were performed using one-way analysis of variance followed by Scheffé's test. *p<0.05; **p<0.01; ***p<0.001 PL: plantaris muscle; CSA: cross-sectional area; I: 10% O₂; II: 15% O₂; III: control; IV: 40% O₂; V: 80% O₂

In the PL, CSA was significantly lower in groups I and II compared to group III by 7.4% and 6.8%, respectively (all p<0.001), whereas groups IV and V exhibited significantly greater CSA than group III by 12.3% and 9.3%, respectively (all p<0.001). Comparisons between high and low O₂ concentrations revealed that CSA in groups I and II was significantly lower than in group IV by 17.6% and 17%, respectively (all p<0.001), and lower than in group V by 15.3% and 14.7%, respectively (all p<0.001) (Figure 4A).

Muscle Type I CSA

The CSA of type I fibers in the Sol was significantly lower in groups I, II, IV, and V than in group III by 17.8%, 21.8%, 7.2%, and 9.7%, respectively (all p<0.001). Under low O₂ conditions, group I showed a significantly greater CSA than group II by 5.2% (p<0.05). When comparing high and low O₂ concentrations, the CSA in groups I and II was significantly lower than that in group IV by 11.4% and 15.8%, respectively (all p<0.001). Conversely, the CSA in group II was significantly lower than in group V by 13.4% (p<0.001) (Figure 3B).

The CSA of type I fibers in the PL was significantly lower in group I than in group III by 9% (p<0.01). Conversely, groups IV and V showed significantly greater CSA than group III by 16.7% (p<0.001) and 8.3% (p<0.05), respectively. Under high O₂ conditions, group IV exhibited significantly higher CSA than group V by 7.8% (p<0.05). When comparing O₂ concentrations, the CSA in groups I and II were significantly lower than in group IV by 22% and 19%, respectively (all p<0.001), and lower than in group V by 15.9% and 12.7%, respectively (all p<0.001) (Figure 4B).

Muscle Type II CSA

The CSA of type II fibers in the Sol was significantly lower in groups I, II, and V than in group III by 13.6%, 17.6%, and 11.5%, respectively (all p<0.001). Under high O₂ conditions, group IV showed a significantly greater CSA than group V by 11.1% (p<0.001). When comparing high and low O₂ concentrations, the CSA in groups I and II was significantly lower than in group IV by 12.2% and 16.2%, respectively (all p<0.001). Moreover, the CSA in group II was significantly lower than in group V by 7% (p<0.001) (Figure 3C).

The CSA of type II fibers in the PL was significantly lower in groups I and II than in group III by 6.5% (p<0.01) and 7.8% (p<0.001), respectively. Conversely, the CSA in groups IV and V was significantly higher than in group III by 9.7% and 10%, respectively (all p<0.001). Comparison between high and low O₂ conditions showed that the CSA in groups I and II was significantly lower than in group IV by 14.7% and 15.9%, respectively (all p<0.001), and also lower than in group V by 14.9% and 16.2%, respectively (all p<0.001) (Figure 4C).

Muscle perimeter

Muscle Total Perimeter

The total perimeter in the Sol was significantly lower in groups I, II, IV, and V than in group III by 5.8%, 9.7%, 3.5%, and 4%, respectively (all p<0.001). Under low O₂ conditions, group I showed a significantly greater perimeter than group II by 4.3% (p<0.001). When comparing O₂ concentrations, the perimeter in groups I and II was significantly lower than in group IV by 2.3% (p<0.05) and 6.3% (p<0.001), respectively. Moreover, the perimeter in group II was significantly lower than in group V by 6% (p<0.001) (Table 1).

**Table 1:** Muscle perimeter in the Sol Statistical analysis were performed using one-way analysis of variance followed by Scheffé's test. Mean±SD; *p<0.05; **p<0.01; ***p<0.001 ^1^: one-way analysis of variance; ^a^: III vs. I; ^b^: III vs. II; ^c^: III vs. IV; ^d^: III vs. V; ^e^: I vs. II; ^f^: I vs. IV; ^g^: I vs. V; ^h^: II vs. IV; ^i^: II vs. V Sol: soleus muscle

Fiber type	Group (O₂ concentration)					F-value	P-value^1^
	I (10% O₂)	II (15% O₂)	III (21% O₂)	IV (40% O₂)	V (80% O₂)		
Total	194.1±35.59^***ae, *f^	186.1±31.00^***behi^	206.1±30.01^***abcd^	198.7±28.51^***ch, *f^	197.9±40.27^***di^	71.21	<0.001
Type I	190.0±25.03^***afg, **e^	184.6±21.24^***bhi, **e^	206.7±22.80^***abcd^	200.1±20.88^***cfh^	200.1±25.25^***dgi^	48.60	<0.001
Type II	199.8±45.81^**e^	188.0±40.11^***b, **e, *h^	205.4±35.92^***b, **c, *d^	197.2±35.12^*eh^	195.6±51.21^**d^	25.84	<0.001

The total perimeter in the PL was significantly higher in groups IV and V than in group III by 8.5% and 4.9%, respectively (all p<0.001). Under high O₂ conditions, group IV exhibited a significantly greater perimeter than group V by 3.5% (p<0.001). In the comparison between O₂ levels, the perimeter in groups I and II was significantly lower than in group IV by 7.8% and 7.9%, respectively (all p<0.001), and also lower than in group V by 4.6% and 4.7%, respectively (all p<0.001) (Table 2).

**Table 2:** Muscle perimeter in the PL Statistical analysis were performed using one-way analysis of variance followed by Scheffé's test. Mean±SD; ***p<0.001 ^1^: one-way analysis of variance; ^a^: III vs. I; ^b^: III vs. II; ^c^: III vs. IV; ^d^: III vs. V; ^e^: I vs. II; ^f^: I vs. IV; ^g^: I vs. V; ^h^: II vs. IV; ^i^: II vs. V; ^j^: IV vs. V PL: plantaris muscle

Fiber type	Group (O₂ concentration)					F-value	P-value^1^
	I (10% O₂)	II (15% O₂)	III (21% O₂)	IV (40% O₂)	V (80% O₂)		
Total	165.3±24.91^***fg^	165.1±21.86^***hi^	165.1±24.27^***cd^	179.2±21.22^***cfhj^	173.2±27.61^***dgij^	55.59	<0.001
Type I	165.8±25.23^***fg^	169.8±26.22^***h^	165.2±26.53^***cd^	185.9±36.44^***cfhj^	175.8±36.44^***dgj^	31.81	<0.001
Type II	164.9±24.72^***fg^	162.1±17.91^***hi^	165.1±22.90^***cd^	171.5±26.97^***cfh^	171.5±28.47^***dgi^	28.70	<0.001

Muscle Type I Perimeter

The perimeter of type I fibers in the Sol was significantly lower in groups I, II, IV, and V than in group III by 8.1%, 10.7%, 3.2%, and 3.2%, respectively (all p<0.001). Under low O₂ conditions, group I had a significantly greater perimeter than group II by 2.9% (p<0.001). When comparing O₂ concentrations, the perimeter in groups I and II was significantly lower than in group IV by 5.1% and 7.8% (all p<0.001), respectively, and also significantly lower than in group V by 5.1% (p<0.01) and 7.8% (p<0.001) (Table 1).

The perimeter of type I fibers in the PL was significantly higher in groups IV and V than in group III by 12.5% and 6.4%, respectively (all p<0.001). Under high O₂ conditions, group IV had a significantly greater perimeter than group V by 5.8% (p<0.001). Comparing O₂ levels, the perimeter in groups I and II was significantly lower than in group IV by 10.8% and 2.4%, respectively (all p<0.001), and the perimeter in group I was significantly lower than in group V by 5.7% (p<0.01) (Table 2).

Muscle Type II Perimeter

The perimeter of type II fibers in the Sol was significantly lower in groups II, IV, and V than in group III by 8.5% (p<0.001), 4% (p<0.05), and 4.8% (p<0.01), respectively. Under low O₂ conditions, group I showed a significantly greater perimeter than group II by 6.3% (p<0.01). Moreover, the perimeter in group II was significantly lower than in group IV by 4.7% (p<0.05) (Table 1).

The perimeter of type II fibers in the PL was significantly higher in groups IV and V than in group III by 6.1% and 3.9%, respectively (all p<0.001). In the comparison between O₂ concentrations, the perimeter in groups I and II was significantly lower than in group IV by 5.9 and 7.5%, respectively (all p<0.001), and also significantly lower than in group V by 3.9% and 5.5%, respectively (all p<0.001) (Table 2).

Oxidative stress and antioxidant abilities

**Figure 5:**
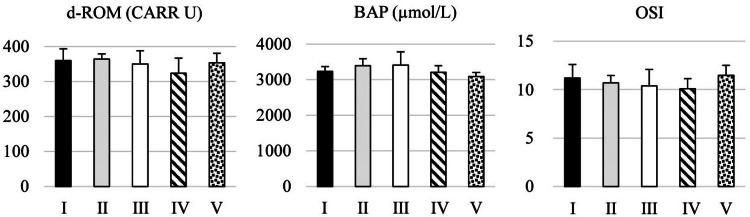
The effects of different oxygen exposures on d-ROM, BAP, and OSI values Statistical analysis were performed using one-way analysis of variance followed by Scheffé's test. I: 10% O₂; II: 15% O₂; III: control; IV: 40% O₂; V: 80% O₂ d-ROM: reactive oxygen metabolite; BAP: biological antioxidant potential; OSI: oxidative stress index

The d-ROM of oxidative stress after the experiment was 323.7 CARR U in group IV to 364.2 CARR U in group II, and the BAP of antioxidative capacity was 3089.3 μmol/L in group V to 3413 μmol/L in group III (Figure 5). The oxidative stress index (OSI), calculated using d-ROM and BAP, ranged from 10.1% in group V to 11.5% in group IV (Figure 5).

## Discussion

This study examined the effects of different O₂ concentrations on muscle tissue and oxidative stress dynamics during the weaning phase. Varying O₂ concentration for one hour during weaning induced fiber type shifting and changes in CSA depending on muscle fiber type characteristics without triggering harmful ROS levels. Additionally, we suggest that a relationship was observed between O₂ concentration and excessive ROS production depending on the duration of exposure.

Capillary density and O₂ diffusion to muscle cells remain higher in type I fibers than in type II fibers [4]. Rich mitochondrial content and elevated oxidative phosphorylation levels characterize type I fibers; therefore, an increased percentage of type I fibers contributes to enhanced O₂ supply and availability [1]. Slot et al. [3] and Matsumoto et al. [5] reported that changes in O₂ concentration increased the percentage of type I fibers in weaning rats. In this study, short-term exposure to low and high O₂ concentrations increased the percentage of type I fibers in the Sol during the weaning phase. Conversion of type II fibers into type I fibers in the Sol results in the proportion of type I fibers reaching approximately 90% in adulthood [23,24], indicating that energy metabolism specificity in the Sol exhibited adaptive responses to O₂ concentration differences. In contrast, the percentage of type I fibers in the PL showed no notable effects from varying O₂ concentrations during short exposure, as type I fibers in the PL eventually transform into type II fibers, comprising approximately 90% [25]. These findings suggested that changes in O₂ concentrations affect muscle fiber types based on the metabolic characteristics of the Sol and PL.

Exposure to low O₂ concentrations during adulthood enhances glycolytic metabolism and leads to a decrease in the percentage of type I fibers and muscle atrophy [12]. In this study, the hypoxic groups I and II showed an increased percentage of type I fibers and decreased CSA in the Sol and PL, corroborating findings from previous studies [2,3]. Negative correlations have been demonstrated between both CSA and oxidative metabolism in muscle tissues, perimeter length, and capillary density [4]. In other words, low O₂ exposure notably activated the fiber-type specificity in the Sol during weaning. These results emphasize the importance of maintaining an adequate O₂ supply to muscle cells. In contrast, hypoxia decreased only the CSA in the PL. Given the high proportion of type II fibers in the PL, low O₂ exposure likely induces muscle atrophy [26]. As muscle atrophy under hypoxic conditions contributes to decreased energy consumption, reduced CSA was observed in both groups I and II. Therefore, short-term exposure to low O₂ concentrations suppressed CSA development and promoted adaptive reactions.

High O₂ concentrations increase dissolved O₂ content in blood serum, promote oxidative metabolism in cells [27], and increase the percentage of type I fibers in diabetes and muscle atrophy [5,6]. Thus, these results indicated that the oxidative metabolism in the Sol was enhanced by short-term exposure to high O₂ concentrations. In contrast to the hypoxic groups, the CSA and perimeter in the PL were increased in the hyperoxic groups. High O₂ promotes protein synthesis and improves muscle atrophy caused by unloading and injuries [6,7]. As CSA in weaning rats increases markedly [28], high O₂ concentrations accelerated muscle development in the PL. Therefore, this study suggested that hyperoxia for one hour promoted the development of muscle fiber type specificity in weaning rats.

ROS, generated during ATP synthesis, plays an essential role in the immune system of organisms [29]. However, excessive ROS production, such as that induced by ischemia and O₂ toxicity, leads to cellular damage. Thus, maintaining the balance between oxidative stress and antioxidant capacity remains essential. Hyperbaric O₂ treatment for stroke and myocardial infarction has been reported to rarely elevate oxidative stress during exposures lasting 60-90 minutes [27]. Due to the immaturity of antioxidant capacity during the weaning phase compared to adulthood, rats at this stage are more susceptible to changes in O₂ concentrations [14]. Deprez et al. [13] reported that exposure to 80% O₂ in newborns increased oxidative stress. In contrast, the one-hour exposure in this study showed no excess production of ROS at any O₂ concentration. This study indicated that varying O₂ concentrations for one hour rarely induced excessive ROS production. Therefore, exposure to low and high O₂ concentrations promoted changes in muscle morphology suited to oxidative metabolism without triggering harmful ROS levels.

Although physical activity accompanied by muscle contraction predominantly increases ROS production, the biological response to O₂ inhalation alone at rest, as observed in this study, remained limited. Varying O₂ concentrations and exercise therapy contribute to advances in developmental physiology, pediatric rehabilitation, and preventive medicine. Future investigations that combine exposure to different O₂ concentrations with exercise loading are expected to provide further insights into the effects of O₂ specificity on muscle morphology and oxidative stress dynamics during weaning. However, one limitation of this study is that we were unable to clarify the effects on oxidative metabolism. Furthermore, considering the small sample size, the influence of varying O₂ concentrations on muscle tissue and oxidative stress dynamics is controversial.

## Conclusions

Exposure to different O₂ concentrations at rest for one hour increased the percentage of type Ⅰ fibers in the Sol during weaning. Particularly, high O₂ concentrations promoted adaptive reactions corresponding to the specificity of muscle fiber types and influenced muscle development during the weaning phase. Additionally, a relationship was observed between O₂ concentration and excessive ROS production depending on the duration of exposure. These findings offer valuable contributions to understanding growth processes and hold promise for applications in developmental physiology. Therefore, our results suggest that varying O₂ concentrations require consideration for clinical applications in pediatric rehabilitation and preventive medicine.

## References

[1] Schiaffino S, Reggiani C (2011). Fiber types in mammalian skeletal muscles. Physiol Rev.

[2] Shin J, Nunomiya A, Kitajima Y, Dan T, Miyata T, Nagatomi R (2016). Prolyl hydroxylase domain 2 deficiency promotes skeletal muscle fiber-type transition via a calcineurin/NFATc1-dependent pathway. Skelet Muscle.

[3] Slot IG, Schols AM, de Theije CC, Snepvangers FJ, Gosker HR (2016). Alterations in skeletal muscle oxidative phenotype in mice exposed to 3 weeks of normobaric hypoxia. J Cell Physiol.

[4] Rivero JL, Talmadge RJ, Edgerton VR (1998). Fibre size and metabolic properties of myosin heavy chain-based fibre types in rat skeletal muscle. J Muscle Res Cell Motil.

[5] Matsumoto A, Nagatomo F, Yasuda K, Tsuda K, Ishihara A (2007). Hyperbaric exposure with high oxygen concentration improves altered fiber types in the plantaris muscle of diabetic Goto-Kakizaki rats. J Physiol Sci.

[6] Ishihara A (2019). Effects of exposure to mild hyperbaric oxygen during unloading on muscle properties in rats. J Muscle Res Cell Motil.

[7] Oyaizu T, Enomoto M, Yamamoto N (2018). Hyperbaric oxygen reduces inflammation, oxygenates injured muscle, and regenerates skeletal muscle via macrophage and satellite cell activation. Sci Rep.

[8] Powers SK, Jackson MJ (2008). Exercise-induced oxidative stress: cellular mechanisms and impact on muscle force production. Physiol Rev.

[9] van Wessel T, de Haan A, van der Laarse WJ, Jaspers RT (2010). The muscle fiber type-fiber size paradox: hypertrophy or oxidative metabolism?. Eur J Appl Physiol.

[10] Magalhães J, Ascensão A, Soares JM, Ferreira R, Neuparth MJ, Marques F, Duarte JA (2005). Acute and severe hypobaric hypoxia increases oxidative stress and impairs mitochondrial function in mouse skeletal muscle. J Appl Physiol (1985).

[11] Boveris A, Chance B (1973). The mitochondrial generation of hydrogen peroxide. General properties and effect of hyperbaric oxygen. Biochem J.

[12] Favier FB, Britto FA, Freyssenet DG, Bigard XA, Benoit H (2015). HIF-1-driven skeletal muscle adaptations to chronic hypoxia: molecular insights into muscle physiology. Cell Mol Life Sci.

[13] Deprez A, Orfi Z, Radu A (2021). Transient neonatal exposure to hyperoxia, an experimental model of preterm birth, leads to skeletal muscle atrophy and fiber type switching. Clin Sci (Lond).

[14] Lee S, Itagaki A, Satoh A, Sugimoto I, Saito T, Shibukawa Y, Tatehana H (2024). Effects of psychogenic stress on oxidative stress and antioxidant capacity at different growth stages of rats: experimental study. PLoS One.

[15] Nagahisa H, Miyata H (2018). Influence of hypoxic stimulation on angiogenesis and satellite cells in mouse skeletal muscle. PLoS One.

[16] Liu W, Pu L, Deng B (2020). Intermittent hypobaric hypoxia causes deleterious effects on the reproductive system in female rats. Biomed Pharmacother.

[17] Beretta E, Lanfranconi F, Grasso GS (2017). Air blood barrier phenotype correlates with alveolo-capillary O2 equilibration in hypobaric hypoxia. Respir Physiol Neurobiol.

[18] Hadanny A, Zubari T, Tamir-Adler L (2019). Hyperbaric oxygen therapy effects on pulmonary functions: a prospective cohort study. BMC Pulm Med.

[19] Levett DZ, Millar IL (2008). Bubble trouble: a review of diving physiology and disease. Postgrad Med J.

[20] Yuba T, Koyama Y, Ootaki C, Fujino Y, Shimada S (2025). Effect of blood sample storage period on d-ROMs and BAP test data. Heliyon.

[21] Lee S, Hashimoto J, Suzuki T, Satoh A (2017). The effects of exercise load during development on oxidative stress levels and antioxidant potential in adulthood. Free Radic Res.

[22] Padykula HA, Herman E (1955). The specificity of the histochemical method for adenosine triphosphatase. J Histochem Cytochem.

[23] Kugelberg E (1976). Adaptive transformation of rat soleus motor units during growth. J Neurol Sci.

[24] Larson L, Lioy J, Johnson J, Medler S (2019). Transitional hybrid skeletal muscle fibers in rat soleus development. J Histochem Cytochem.

[25] Whalen RG, Johnstone D, Bryers PS, Butler-Browne GS, Ecob MS, Jaros E (1984). A developmentally regulated disappearance of slow myosin in fast-type muscles of the mouse. FEBS Lett.

[26] Wüst RC, Jaspers RT, van Heijst AF, Hopman MT, Hoofd LJ, van der Laarse WJ, Degens H (2009). Region-specific adaptations in determinants of rat skeletal muscle oxygenation to chronic hypoxia. Am J Physiol Heart Circ Physiol.

[27] Ishihara A (2019). Mild hyperbaric oxygen: mechanisms and effects. J Physiol Sci.

[28] White RB, Biérinx AS, Gnocchi VF, Zammit PS (2010). Dynamics of muscle fibre growth during postnatal mouse development. BMC Dev Biol.

[29] Powers SK, Ji LL, Leeuwenburgh C (1999). Exercise training-induced alterations in skeletal muscle antioxidant capacity: a brief review. Med Sci Sports Exerc.

